# Prevalence, awareness, treatment and control of young-onset hypertension in Malaysia, 2006–2015

**DOI:** 10.1038/s41371-020-00478-0

**Published:** 2021-02-03

**Authors:** Yi Yi Khoo, Nik Daliana Nik Farid, Wan Yuen Choo, Azahadi Omar

**Affiliations:** 1 Department of Social and Preventive Medicine, Faculty of Medicine, University of Malaya, Kuala Lumpur, Malaysia; 2 Centre for Population Health, Faculty of Medicine, University of Malaya, Kuala Lumpur, Malaysia; 3 Biostatistics and Data Repository, National Institutes of Health, Ministry of Health, Selangor, Malaysia

**Keywords:** Hypertension, Preventive medicine, Diagnosis, Risk factors

## Abstract

The continuous presence of elevated blood pressure (BP) when young is a strong predictor of future cardiovascular risk. This study aimed to elucidate the prevalence, awareness, treatment and control of young-onset hypertension (YOH) in Malaysia during the period 2006–2015. Data on respondents aged 18–39 diagnosed with YOH according to the 7th Joint National Committee Report (USA) were extracted from three National Health and Morbidity Surveys (2006, 2011, and 2015). The prevalence of YOH remained stable: 17.7%, 95% CI [17.0, 18.3] in 2006, 17.0%, 95% CI [16.0, 17.9] in 2011 and 18.4%, 95% CI [17.4, 19.4] in 2015. Awareness, treatment and control rates were suboptimal; 15% were aware of their diagnosis, of which less than 50% were on treatment and less than 40% who were on treatment had their BP controlled. Trend analysis revealed a significant increase in YOH prevalence among urban dwellers; those with no formal and tertiary education and middle-income earners. YOH awareness and treatment rates were lower among respondents <30 years; however, when treated, this group achieved overall better control rates. Females had higher awareness and treatment rates, but lower control. Treatment rates remained stable for all ethnicities with the exception of Chinese, which decreased. This study narrows the knowledge gap on YOH epidemiology in Malaysia by providing crucial information on the pervasiveness of hypertension among young adults. Results can be used to develop non-communicable disease policies and health promotion strategies specially targeted at young adults who are in the prime of life.

## Introduction

Globally, over a period of almost three decades from the 1990s as agrarian-based societies transition towards industrialization, cardiovascular diseases (CVDs) such as ischaemic heart disease and stroke have risen in ranks to be among the top five leading causes of global burden of disease, surpassing the traditional contributors such as lower respiratory infections and diarrhoeal diseases [1]. Many prospective epidemiological studies have shown that the continuous presence of elevated blood pressure (BP) has an adverse effect on the risk of developing future cardiovascular events [2]. Recognizing the significance of this, several communities and countries have started to investigate hypertension among young adults in order to determine the pervasiveness of this problem in their local setting. In a national study in the USA, it has been found that 7.3% of young adults aged 18–39 had hypertension during the period 2013–2014 [3]. Results from Asia and Africa varied widely—with prevalence rates as low as 1.6% in Singapore among men aged 17–23 years [4] to 15% among young adults aged 18–40 years in Uganda [5]. Awareness, treatment and control rates were vastly different as well in the aforementioned studies in the USA and Africa (awareness: 74.7% vs. 13.7%; treatment: 50% vs. 100%; control 80.4% vs. 19.7%) [4, 5]. In Malaysia however, young adults as a population group have largely been overlooked by researchers perhaps because the approach to addressing hypertension seems to be one that focuses on treating the later consequences of CVDs [6]. Furthermore, Malaysia’s current healthcare system is still very much focused on curative rather than preventive care, with 70% of service expenditure allocated to therapeutic care and only 5% to public health services (which include health promotion and prevention programmes) [7]. This justified the need to conduct a study on young-onset hypertension (YOH) in Malaysia to ascertain the burden in the context of an upcoming developing nation. The aim of this study is to elucidate the prevalence, awareness, treatment and control of YOH in Malaysia, and examine the trend for a decade from 2006 to 2015. This study contributes to the limited but growing literature surrounding the epidemiology of YOH and is the first few of its kind using a nationally representative, population-based study to focus on hypertension among young adults.

## Materials and methods

This study was approved by the Medical Research and Ethics Committee (MREC), Ministry of Health, Malaysia (NMRR-19-2045-49266).

### Study design and population

Situated in Southeast Asia, Malaysia consists of two regions (East and West) separated by the South China Sea. Secondary data analysis of decade long data from three Malaysian National Health and Morbidity Surveys (NHMSs) 2006, 2011 and 2015 were utilized. The NHMS is commissioned by the Ministry of Health, Malaysia, to provide community-based data on the patterns of common health problems, health service utilization and health expenditures of the population. A cross-sectional study design was employed, using a two-stage stratified random sampling selection throughout Malaysia provided by Department of Statistics Malaysia (DOSM). The first stage sampling unit was the enumeration block and, within each sampled block, living quarters (LQs) were selected as the second stage. All households and eligible persons within each selected LQ were included in the study. The total samples in each cohort were 56,710, 26,498 and 29,460 respondents for NHMSs 2006, 2011 and 2015, respectively. The final analysis was based on 16,565, 8678 and 8906 young adults who satisfied the following criteria.

Inclusion criteria:

1. Any individual aged 18–39 within the selected LQs.
2. Individuals living in Malaysia for at least 2 weeks prior to data collection.

Exclusion criteria:

1. Institutionalized populations for example those residing in hotels, hospitals, prisons, etc.
2. Pregnant women.

In the local Malaysian context and for the purposes of this study, young adults are defined as those aged 18–39 years inclusive. The minimum age of 18 was selected because the NHMSs consider those aged ≥18 years to be adults. The cut-off of <40 years was based on the use of similar age strata used by several other relevant works on young adults [3, 8], thus allowing comparisons with other studies to be made.

### Data collection

NHMS data were collected by trained research team members using structured questionnaires, clinical examination and biochemical analyses. Informed written consent from the respondents is a requirement for participation in all NHMSs.

#### Blood pressure

The average measurements of two readings of systolic and diastolic BPs taken 15 min apart in a sitting position by trained nurses with validated and calibrated Omron Digital Automatic BP Monitor Model HEM-907 was taken into account. The hypertension definition used in this study were individuals who had an average SBP of 140 mmHg and/or an average DBP of 90 mmHg or greater, or was previously informed by medical personnel to have hypertension, as classified by *The Seventh Report of the Joint National Committee on Prevention, Detection, Evaluation, and Treatment of High Blood Pressure* (JNC 7) [9]. Awareness was defined as answering “yes” to the question “Have you ever been told by a doctor or paramedic that you have raised BP or hypertension?”. Treatment of hypertension was defined as self-report of respondents taking antihypertensive medication, or receiving advice for hypertension (lose weight, reduce salt intake, start/do more exercise). Controlled hypertension was defined as having a desirable BP level (<140/<90 mmHg) among those with hypertension.

#### Sociodemographic factors

Age was grouped into <30 and ≥30 years. Locality was categorized as either rural or urban based on DOSM definition. Ethnicity was categorized as “Malay” (majority ethnic group in Malaysia), “Chinese”, “Indian”, “Indigenous peoples” or “Others” (respondents who belonged to other than the aforementioned four ethnicities). Examples of “Indigenous peoples” include the Iban, Bajau and Kadazan/Dusun tribes from West Malaysia states of Sabah and Sarawak. Examples of respondents who belonged to the “Others” category include Eurasian, Indonesian, Thai and Filipino ethnicities. Education was categorized as “no formal/none”, primary, secondary, tertiary or unclassified (other education level not fitting into the aforementioned categories). Income was defined according to DOSM Household Income and Basic Amenities survey categories of T20 (top 20%) with a median household income of ≥RM 13,148; M40 (middle 40%) with a median household income of ≥RM 6275 or B40 (bottom 40%) with a median household income of ≥RM 3000, otherwise colloquially termed as upper, middle and lower classes [10].

### Statistical analyses

Patterns of missing data were assessed and determined to be missing not at random as responses on key items differed greatly. Missing data (3.1%) (variables: education level, household income and YOH) were remodelled with the multiple imputation technique using Stata Statistical Software v.15 (StataCorp, USA). Subsequently, data from the NHMS 2006, 2011 and 2015 were analysed using the complex sample module in Statistical Package for Social Sciences Program Version 20.0 (IBM, USA). Sampling weights were used to adjust for non-response bias. The analysis was conducted to ensure design effect and sample weights were accounted for. The age-adjusted rates of YOH prevalence, awareness, treatment and control were computed using 2010 Census Data, which allowed comparisons across different survey cycles. Multivariate logistic regression analysis was performed with YOH as the outcome variable to examine the effects of the sociodemographic factors (gender, age group, ethnicity, locality and socioeconomic status) as potential independent risk factors for YOH. A comparison of findings is possible as all NHMS surveys used a similar methodology. Further details on NHMS methodology is found elsewhere [11].

## Results

### Characteristics of the study population

The characteristics of the study population are presented in Table 1. Over half of the respondents in all three surveys were female. Approximately 60% of participants were aged <30 and resided in urban localities. The majority ethnic group for each survey comprised of Malays. More than half of the respondents attained secondary level education and more than three quarters of the study population were low income earners.

**Table 1:** Characteristics of the study population (young adults aged 18–39), NHMS 2006–2015.

NHMS year (*N* = 34,149)							
Variables		2006 (*N* = 16,565)		2011 (*N* = 8678)		2015 (*N* = 8906)	
		*N*	%	*N*	%	*N*	%
Gender	Male	7318	44.2	4129	47.6	4352	48.9
	Female	9247	55.8	4549	52.4	4554	51.1
Age group	<30	10,064	60.8	5406	62.3	5330	59.8
	≥30	6501	39.2	3272	37.7	3576	40.2
Locality	Urban	10,229	61.8	5287	60.9	5459	61.3
	Rural	6336	38.2	3391	39.1	3447	38.7
Ethnicity	Malays	9147	55.2	5102	58.8	5519	62.0
	Chinese	2650	16.0	1271	14.6	1085	12.2
	Indians	1369	8.3	649	7.5	620	7.0
	Indigenous peoples	2162	13.1	900	10.4	871	9.8
	Others	1237	7.5	756	8.7	811	9.1
Education	None	438	2.6	152	1.8	202	2.3
	Primary	3477	21.0	992	11.4	937	10.5
	Secondary	10,086	60.9	4629	53.4	4620	51.9
	Tertiary	2364	14.3	2788	32.1	3002	33.7
	Unclassified	200	1.2	117	1.3	144	1.6
Household income	B40	14,705	88.8	6149	70.9	5662	63.6
	M40	1546	8.3	2123	24.5	2543	28.6
	T20	314	1.9	406	4.7	701	7.8

### Prevalence of YOH

**Fig. 1:**
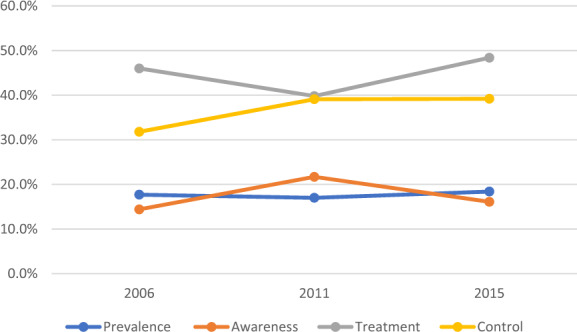
Age-standardized weighted prevalence, awareness, treatment, and control rates of young-onset hypertension in Malaysia, 2006–2015. Prevalence of YOH (blue) remained stable affecting nearly 2 million young adults for every survey year. Awareness, treatment and control rates were suboptimal; 15% were aware of their diagnosis (orange), of which less than 50% were on treatment (grey) and less than 40% who were on treatment had their BP controlled (yellow).

Throughout the decade, the age-adjusted prevalence of YOH in Malaysia was 17.7%, 95% CI [17.0, 18.3] in 2006, 17.0%, 95% CI [16.0, 17.9] in 2011 and 18.4%, 95% CI [17.4, 19.4] in 2015 (Table 2 and Fig. 1). There was an obvious male preponderance (ratio 1.5:1) of YOH. YOH was twice as common among those aged ≥30 years compared to adults aged <30 years. Trend analysis for age and gender remained stable throughout the decade. YOH was most prevalent among ‘Indigenous peoples’ in all three surveys, whereas Chinese and Indians had the lowest prevalence rates. In general, YOH trends for age, gender and all racial groups were stable between 2006 and 2015. Although the prevalence of YOH decreased in the rural population from 2006 to 2015 compared to urban dwellers, this group still displayed a higher overall prevalence of YOH. Interestingly, however, the rate of change from 2006 to 2015 was more pronounced in the urban community, a trend which was statistically significant. Throughout the decade, prevalence of YOH was lowest among those who achieved tertiary education. The proportion of hypertensives was lowest among young adults with an upper-class income (T20 earners). There was an increasing trend in YOH over the 10-year period in all income groups; however, it was only statistically significant for the middle-income earners.

**Table 2:** Prevalence of YOH by sociodemographic risk factors, NHMS 2006–2015.

Year	2006 (*N* = 16,287)			2011 (*N* = 8612)			2015 (*N* = 8906)				
	*n*	Estimated population	% (95% CI)	*n*	Estimated population	% (95% CI)	*n*	Estimated population	% (95% CI)	Change 2006–2015, % (95% CI)	Odds ratio (OR) (2006–2015)
Overall	2943	1,937,274	17.7 [17.0, 18.3]	1507	1,861,730	17.0 [16.0, 17.9]	1699	2,017,955	18.4 [17.4, 19.4]	0.7 [0.4, 1.1]	1.05 [0.97, 1.14]
Sociodemographic factors											
Gender											
Male	1590	1,228,767	21.4 [20.4, 22.5]	844	1,153,626	20.1 [18.8, 21.5]	969	1,248,683	21.8 [20.4, 23.2]	0.4 [0.0, 0.7]	1.02 [0.92, 1.13]
Female	1353	708,506	13.5 [12.8, 14.3]	663	708,104	13.5 [12.5, 14.7]	730	769,171	14.7 [13.6, 15.8]	1.2 [0.8, 1.5]	1.10 [0.99, 1.23]
Age group											
<30	1333	1,014,717	13.9 [13.2, 14.7]	675	921,872	12.6 [11.6, 13.7]	779	1,063,051	14.6 [13.5, 15.7]	0.7 [0.3, 1.0]	1.06 [0.94, 1.18]
≥30	1610	922,557	25.0 [23.8, 26.1]	832	939,858	25.7 [24.2, 27.3]	920	954,903	25.9 [24.4, 27.4]	0.7 [0.6, 1.3]	1.05 [0.95, 1.16]
Locality											
Urban	1597	1,055,853	15.6 [14.8, 16.5]	888	1,097,883	16.5 [15.3, 17.7]	982	1,171,573	17.5 [16.3, 18.7]	1.9 [1.5, 2.2]	1.15 [1.03, 1.27]^a^
Rural	1346	881,421	20.9 [19.8, 22.1]	619	763,847	17.7 [16.3, 19.3]	717	846,381	19.8 [18.2, 21.5]	−1.1 [−1.6, −0.6]	0.93 [0.83, 1.06]
Ethnicity											
Malay	1681	1,110,063	18.2 [17.4, 19.1]	903	1,111,610	17.2 [16.0, 18.4]	1061	1,260,622	18.5 [17.3, 19.7]	0.3 [−0.1,0.6]	1.02 [0.92, 1.12]
Chinese	399	264,902	15.3 [13.8, 16.9]	191	239,578	15.1 [13.2, 17.3]	165	196,044	14.9 [12.8, 17.3]	−0.4 [−1.0, 0.4]	0.97 [0.78, 1.20]
Indian	196	128,289	14.2 [12.4, 16.2]	100	124,350	15.2 [12.6, 18.3]	101	119,583	15.6 [12.7, 19.0]	1.4 [0.3, 2.8]	1.16 [0.84, 1.48]
Indigenous peoples	441	286,171	20.1 [18.3, 22.2]	195	246,089	21.5 [18.8, 24.6]	206	244,390	22.9 [20.0, 26.1]	2.8 [1.7, 3.9]	1.18 [0.95, 1.45]
Others	226	147,847	17.9 [15.4, 20.7]	118	140,102	14.7 [12.0, 17.8]	166	197,313	19.8 [16.5, 23.6]	1.9 [1.1, 2.9]	1.14 [0.85, 1.51]
Education level											
None	100	64,014	23.7 [19.8, 28.0]	27	33,238	17.8 [12.2, 25.4]	34	40,840	16.2 [11.3, 22.6]	−7.5 [−8.5, 5.4]	0.62 [0.39, 0.99]^a^
Primary	696	439,330	19.5 [18.2, 21.0]	208	246,077	20.0 [17.6, 22.5]	241	274,239	25.0 [22.1, 28.1]	5.5 [3.9, 7.1]	1.37 [1.14, 1.65]^a^
Secondary	1806	1,196,389	17.8 [17.0, 18.7]	854	1,051,677	17.9 [16.7, 19.2]	886	1,048,806	18.2 [17.0, 19.5]	0.4 [0.0, 0.8]	1.03 [0.93, 1.13]
Tertiary	303	1,604,449	13.6 [12.1, 15.2]	407	516,560	14.7 [13.3, 16.2]	510	618,716	16.8 [15.4, 18.3]	3.2 [3.3, 3.1]	1.29 [1.09, 1.52]^a^
Unclassified	30	19,565	14.4 [10.3, 19.7]	11	14,178	8.9 [4.7, 16.0]	28	35,342	19.0 [13.3, 26.3]	4.6 [3.0, 6.6]	1.39 [0.79, 2.45]
Household income											
B40	2688	1,763,700	18.1 [17.4, 18.8]	1121	1,381,005	17.7 [16.7, 18.8]	1147	1,349,486	19.3 [18.1, 20.5]	1.2 [0.7, 1.7]	1.08 [0.99, 1.19]
M40	218	147,811	14.5 [12.8, 16.5]	338	421,360	15.7 [14.0, 17.5]	438	533,719	17.1 [15.5, 18.8]	2.6 [2.7, 2.3]	1.21 [1.01, 1.47]^a^
T20	37	25,762	12.7 [8.2, 19.1]	48	59,365	11.7 [8.8, 15.4]	114	134,749	15.8 [13.1, 18.8]	3.1 [4.9, 9.7]	1.29 [0.76, 2.19]

^a^significant OR

### Awareness, treatment and control of YOH

In 2015, 16.1%, 95% CI [14.3, 18.1] of young adults with YOH were aware of their diagnosis, with 48.4%, 95% CI [42.6, 54.2] on treatment (of which 98.1% were given both lifestyle advice and medication, and 1.9% were prescribed medication), and BP was controlled in 39.2%, 95% CI [31.3, 47.6] among those who were on treatment. Awareness and treatment rates remained stable from 2006 to 2015, at 1.7% (*p* trend = 0.397) and 2.4% (*p* trend = 0.662), respectively. There was a non-statistically significant increase of controlled BP rates (among those who were on treatment) by 7.4% (*p* = 0.179) from 2006 to 2015 (Table 3 and Fig. 1).

**Table 3:** Prevalence of awareness, treatment and control of YOH, NHMS 2006–2015.

	Prevalence (%)			Change, 2006–2015	
NHMS year	2006	2011	2015	%	*p* value
Awareness					
Among all hypertensives	14.4 [13.1, 15.8]	21.7 [19.7, 24.0]	16.1 [14.3, 18.1]	1.7 [1.2, 2.3]	0.397
Treatment					
Among those who are aware	46.0 [41.2, 50.9]	39.8 [34.7, 45.1]	48.4 [42.6, 54.2]	2.4 [1.4, 3.3]	0.662
Control					
Among those treated	31.8 [25.8, 38.5]	39.1 [31.1, 47.7]	39.2 [31.3, 47.6]	7.4 [5.5, 9.1]	0.179

The separate effects of age, sex and ethnicity on YOH awareness, treatment and control were examined (Table 4). Although awareness of YOH increased significantly among young adults aged < 30 years throughout the decade; overall, awareness rates were still lower in this group compared to those ≥30 years. A similar pattern was seen in YOH treatment rates. However, among young adults who were on treatment for YOH, better control was achieved in the younger age group than in the older age group. Females generally showed higher awareness and treatment rates of their disease compared to males, but lower control rates. YOH awareness rates were unchanged in all racial/ethnic groups between 2006 and 2015. Treatment rates also remained stable for all ethnic groups with the exception of Chinese which decreased. Control rates improved by at least 20% for almost all ethnicities with the exception of Malays which remained stable.

**Table 4:** Awareness, treatment and control of YOH by age, sex and ethnicity, 2006–2015.

NHMS year	Prevalence			Change 2006–2015	Odds ratio
	2006	2011	2015	% (95% CI)	2006–2015
Awareness					
Age					
<30	9.2 [7.7, 11.0]	19.0 [16.3, 22.0]	13.6 [11.4, 16.2]	4.4 [3.7, 5.2]	1.56 [1.17, 2.07]^a^
≥30	20.1 [18.1, 22.2]	24.4 [21.6, 27.5]	19.0 [16.3, 21.9]	−1.1 [−1.8, −0.3]	0.93 [0.75, 1.16]
Gender					
Male	11.9 [10.3, 13.7]	18.9 [16.4, 21.7]	13.8 [11.7, 16.3]	1.9 [1.4, 2.6]	1.19 [0.93, 1.53]
Female	18.7 [16.7, 20.9]	26.4 [23.1, 30.0]	19.9 [17.1, 23.0]	1.2 [0.4, 2.1]	1.08 [0.86, 1.36]
Ethnicity					
Malays	14.0 [12.3, 15.8]	23.6 (20.9, 26.5]	15.3 [13.2, 17.7]	1.3 [0.9, 1.9]	1.11 [0.89, 1.40]
Chinese	14.1 [10.9, 18.2]	19.5 (14.5, 25.6]	16.6 [11.5, 23.4]	2.5 [0.6, 5.2]	1.21 [0.72, 2.05]
Indians	16.8 [11.9, 23.2]	16.7 (10.6, 25.2]	22.5 [15.2, 32.0]	5.7 [3.3, 8.8]	1.44 [0.77, 2.71]
Indigenous peoples	17.1 [13.9, 20.8]	22.4 (16.8, 29.3]	22.3 [16.7, 29.2]	5.2 [2.8, 8.4]	1.40 [0.91, 2.16]
Others	10.8 [7.4, 15.4]	14.3 (9.1, 21.8]	9.4 [5.8, 15.0]	−1.4 [−1.6, −0.4]	0.87 [0.44, 1.69]
Treatment					
Age					
<30	31.6 [23.7, 40.8]	27.8 [20.8, 36.2]	30.7 [22.6, 40.3]	−0.9 [−1.1, −0.5]	0.96 [0.54, 1.71]
≥30	53.2 [47.6, 58.8]	48.9 [42.0, 55.8]	62.5 [54.7, 69.7]	9.3 [7.1, 10.9]	1.46 [0.99, 2.1]
Gender					
Male	40.3 [33.6, 47.4]	35.5 [28.5, 43.0]	48.0 [39.5, 56.6]	7.7 [5.9, 9.2]	1.37 [0.87, 2.14]
Female	52.3 [46.0, 58.5]	44.8 [37.6, 52.3]	48.8 [41.0, 56.8]	−3.5 [−5.0, −1.7]	0.87 [0.58, 1.31]
Ethnicity					
Malays	43.8 [37.5, 50.4]	41.3 [35.0, 47.9]	49.9 [42.7, 57.0]	6.1 [5.2, 6.6]	1.28 [0.86, 1.89]
Chinese	64.3 [50.4, 76.2]	38.6 [24.4, 55.1]	42.8 [25.8, 61.8]	−21.5 [−24.6, −14.4]	0.42 [0.16, 1.09]
Indians	49.8 [33.9, 65.7]	41.2 [20.5, 65.5]	56.6 [36.4, 74.8]	6.8 [2.5, 9.1]	1.32 [0.26, 3.78]
Indigenous peoples	41.6 [30.9, 53.2]	34.4 [22.1, 49.2]	43.0 [28.5, 58.8]	1.4 [−2.4, 5.6]	1.06 [0.48, 2.34]
Others	32.8 [17.6, 52.7]	36.6 [19.1, 58.6]	46.8 [25.1, 69.8]	14.0 [7.5, 17.1]	1.80 [0.51, 6.43]
Control					
Age					
<30	38.2 [24.1, 54.6]	49.1 [33.5, 64.9]	49.4 [32.3, 66.6]	11.2 [8.2, 12.0]	1.58 [0.60, 4.20]
≥30	29.9 [23.6, 37.1]	34.8 [25.9, 44.8]	35.1 [26.7, 44.6]	5.2 [3.1, 7.5]	1.27 [0.76, 2.11]
Gender					
Male	34.0 [24.4, 45.5]	37.8 [26.5, 50.5]	41.0 [29.2, 53.9]	7.0 [4.8, 8.4]	1.35 [0.66, 2.73]
Female	30.0 [23.0, 38.0]	40.3 [30.0, 51.7]	37.1 [26.5, 49.2]	7.1 [3.5, 11.2]	1.38 [0.75, 2.54]
Ethnicity					
Malays	29.4 [21.5, 38.8]	33.6 [24.5, 44.1]	30.9 [21.9, 41.6]	1.5 [0.4, 2.8]	1.07 [0.57, 2.01]
Chinese	38.3 [24.4, 54.4]	62.6 [39.0, 81.5]	61.4 [34.0, 83.1]	23.1 [9.6, 28.7]	2.57 [0.70, 9.44]
Indians	44.4 [24.8, 65.9]	30.8 [7.8, 69.9]	67.1 [40.3, 86.0]	22.7 [15.5, 20.1]	2.56 [0.62, 10.51]
Indigenous peoples	28.7 [16.9, 44.3]	53.1 [27.3, 77.4]	49.7 [27.4, 72.1]	21.0 [10.5, 27.8]	2.46 [0.76, 7.99]
Others	20.4 [4.9, 55.9]	36.3 [9.9, 74.8]	17.6 [2.5, 63.5]	−2.8 [−2.4, 7.6]	0.83 [0.06, 11.65]

## Discussion

Our results demonstrate that in a representative sample of noninstitutionalised young Malaysian adults, the age-adjusted prevalence of YOH remained unchanged for a decade from 2006 to 2015, affecting almost 20% of the study population—which equates to ~2 million young Malaysian adults being diagnosed with YOH (Table 2). Prevalence of YOH in Malaysia is comparable to other developing countries such India [12] and Indonesia [13], but higher than in developed countries, such as the USA [3]. The stable prevalence of YOH prevalence for a decade is consistent with other studies that investigated the trend/time-series of YOH [3]. Since the beginning of the 21st century, the world has seen large investments in maternal and child health causing great improvements for early years of life [14], followed by heavily skewed allocations towards the control of infectious diseases [15], but generally a low global investment in non-communicable diseases (NCDs). A similar situation is mirrored here in Malaysia with a primary healthcare system that prioritizes the provision of maternal and child healthcare and infectious diseases management, but not NCDs [6]. The situation is compounded as “young invincibles” may consider themselves invulnerable towards developing chronic diseases [16], hence they do not readily seek routine screening or treatment. The combination of these two factors, that is, the lack of attention by healthcare systems towards NCDs and young adults’ insouciant attitudes towards their own health, cumulatively poses a challenge for public health professionals.

The prevalence of YOH increased significantly among urban dwellers over the 10 years covered by this study. This could be due to the increasing numbers of urban–urban and rural–urban migrants during these years, with currently 77.8% of the Malaysian population now living in urban areas [17]. Young adults face health challenges that may be caused in part by rapid urbanization as cities grow to support faster-paced lifestyles, as evidenced by more available types of transportation and the proliferation of fast food outlets. Hence, the selection of fast and unhealthy food options becomes normalized for young people living in cities. Indeed, studies conducted among young adults in cities in Africa, the Middle East and Asia have shown that lifestyle behaviours such their dietary choice [18], increasingly sedentary lifestyles [19] and smoking [12] were predictive of YOH in their study population.

Prevalence of YOH among the bottom 40% (lower income) earners was consistently the highest compared with other income groups. However, interestingly, the prevalence of YOH increased significantly among the middle-income group at a statistically significant rate. A recent 2018 Malaysian State of Households Survey revealed that since the 1970s, there has been a gradual increase in the gaps between the upper, middle and lower income groups [20]. As part of the 11th Malaysia Plan in “uplifting B40 (lower income) households towards a middle-class society”, specific financial programmes were targeted for this group; for example, free health protection insurance for 36 critical illness and income replacement due to hospitalization, incentives for completing NCDs/cancer treatments for B40 recipients aged 50, cash transfers aid to cushion the rising cost of living, RM1 billion fund to finance the purchase of their first home as well as targeted fuel subsidies [21]. As most of the financial aid goes to the lower income group, the middle-income earners—majority of whom live in the city and include mostly salary earners in both the private and public sectors—may be experiencing the ongoing frustration of having to deal with a declining disposable income and the increasing costs to maintain a comfortable standard of living. These results indicate that both the lower- and middle-income groups should be targeted for YOH prevention and awareness.

Our results reveal that less than half of young adults in all 3 survey years were on some form of treatment for hypertension, whether lifestyle advice or medication. These findings could be attributed to the decisional ambiguity by healthcare professionals regarding the advantages vs. disadvantages of early pharmacological intervention in this age group [3]. Further, there is evidence that hypertensive young adults are less likely to receive advice on lifestyle changes to curtail hypertension, such as healthy diet and exercise [22]. As lifestyle behaviours have been shown to be a significant determinant of YOH [12, 18, 19], healthcare professionals should work with individuals in a variety of settings to adapt their choices to develop healthy behaviours tailored to accommodate physical health, cultural, ethnic, traditional and personal preferences, as well as personal food budgets and other issues of accessibility. Previous research has demonstrated that sustainable lifestyle behavioural change is more likely when these modifications are integrated into one’s personality and internally motivated [23].

This study analysed the separate effects of several sociodemographic variables such as age, gender and ethnicity on awareness, treatment and control rates (Table 4). In general, YOH prevalence, awareness and treatment rates were lower among those aged < 30 compared to their older counterparts. Yahia et al. conducted an assessment among 243 college students on their awareness and knowledge about conditions relevant to metabolic syndrome, and reported that less than half of their young adult study population were aware of the relationship between hypertension and arteriosclerosis [24]. Nonetheless, among this younger group, awareness increased at a significant rate over the decade 2006–2015. The reason behind this is may be due to improved health vigilance. The 2015 Nielsen Global Health and Wellness Survey found that consumers around the world are attempting to take charge of their health by making more healthful food choices by, for example, reducing their intake of sugar, cholesterol, trans and saturated fats and sodium [25]. Of note, higher rates of controlled BP were achieved in the younger age group among those who were being treated, consistent with other studies [3]. These observations suggest that improving the healthcare system to better prevent and detect hypertension among young adults may improve BP control in Malaysia. In addition, improving YOH awareness should result in improved treatment and control rates.

The subgroup analysis revealed gender-based disparities in YOH awareness, treatment and control, similar to those observed in other studies [3]. Gender-related differences were prominent in this study, with men having a higher prevalence of YOH compared to women throughout the decade, results which were consistent with literature pertaining to YOH [12, 13]. Studies have shown that on average, young females recorded more regular healthcare visits compared to their male counterparts [3], which increases the likelihood of having their BP examined than young men [26]. Despite having better awareness and treatment rates compared to young adult men, BP control rates of young adult women who were on treatment (whether lifestyle advice or medication) were slightly lower than their counterparts, contrasting with results from other studies [3]. There could be several reasons for this disparity in results. First, with lifestyle modification alone, there is evidence that BP control is worse in women than in men [27]. Next, with regard to medication use, the female gender may be a risk factor for adverse effects or diminished clinical responses to antihypertensive drugs [28], which may in turn lead to poorer treatment compliance and hence poorer BP control in the long term. These findings suggest that a customised approach is necessary to select an optimal therapeutic plan that effectively lowers BP (which includes a multifaceted approach to therapy using both lifestyle modification and pharmacotherapy), prevents CVD and minimizes adverse effects in women.

Dietary intake may also play a role in influencing ethnic variations in YOH epidemiology. Previous studies have consistently shown that, in Malaysia, the indigenous peoples of Sarawak and Sabah (comprising of many different native tribes as described earlier) had the highest sodium intake as assessed using methods such as the food frequency questionnaire and 24-h dietary recall [29], which could explain the findings in our study of the highest YOH prevalence rate in this group. In a study conducted in the USA, hypertension awareness was found to be doubled among young adults who had regular visits to a healthcare provider over the last 2 years [30]. This could explain the difference in the YOH awareness rates between the ethnicities in 2015 whereby the indigenous peoples of Sarawak and Sabah had the highest outpatient care utilization rates, while “Others” had the lowest [31]. According to the analysis of the data in the three NHMSs, hypertension treatment rates for the Chinese ethnic group decreased from 2006 to 2015. This may be due in part to Chinese resorting to other modes of treatment such as traditional and complementary medicine (T&CM); indeed, in 2015, Chinese self-reported the highest prevalence ever of the use of T&CM [32]. The usage of T&CM, including supplements and health foods, has been shown to be correlated with lower compliance to antihypertensive medication [33].

This study obtained secondary data from three NHMSs which had been conducted among a representative sample of the Malaysian adult population using standard protocols. The strengths of the NHMS included a high response rate, two BP measurements in the same visit by trained medical personnel and detailed information on history of hypertension and pharmaceutical treatment. Strict training processes for data collectors were implemented and validated questionnaires and instruments were employed to ensure the quality of the data. Ten years of data collected over three national surveys and the large sample size allowed high precision estimation of age-specific trends. Furthermore, weights were applied and age adjustment was performed according to the 2010 Malaysian population census. Hence, the findings of the current study are generalizable and provide the most reliable and up-to-date information on the current trend in YOH in the Malaysian population.

It is also recognized that missing data are an inherent problem in the analysis of secondary data. To address this drawback, all variables were systematically reviewed and missing data were rectified using statistical methods such as the multiple imputation technique in order to minimize as much as possible the effect of missing data on the results. As regards the measures themselves, as BP was measured only during a single visit, the prevalence of hypertension may have been overestimated. Nonetheless, the use of BP measurements taken during one visit is a well-recognized inherent problem of large epidemiological investigations and should have minimal effects on within-sample comparisons.

The study revealed that that the prevalence of YOH in Malaysia was comparable to that seen in other developing countries and that this prevalence had remained stable from 2006 to 2015, affecting nearly one fifth of the young adult population. The analysis also showed that awareness, treatment and control rates were suboptimal over the 10-year period. These findings indicate that YOH has been a significant public health problem for the past decade, and will only continue to remain a problem unless major strides are undertaken. The increasing prevalence of YOH among the urban population and those of differing socioeconomic status highlights the need for interventions that would target prevention in these groups. Public health preventive measures should focus on the younger age group as evidence shows that when they are treated, they are more likely to achieve better overall control. Among men, the emphasis should be on improving their awareness and treatment rates, whereas in women, the focus should be on measures to improve persistence in treatment adherence. The different sociocultural beliefs and values affecting different races should be considered during consultations as they may have substantial impact on YOH treatment adherence and control. Furthermore, the adoption of the new 2017 American College of Cardiology/American Heart Association definition of hypertension and treatment target from 140/90 to 130/80 mmHg is expected to lead to a substantial increase in the prevalence of YOH with BP levels above the treatment goals, thus indicating that addressing hypertension at a younger age may help prevent the complications of hypertension [34].

The influence of cultural factors across ethnic populations cannot be ignored in respect of addressing the issue of adherence to medication compliance and lifestyle changes, especially in a multiracial country such as Malaysia. It would be worthwhile to undertake further research to identify the genetics contributing to these disparities and explore how healthcare utilization uptakes and treatment adherence differ across these ethnicities [35]. Throughout the process of conducting this study, defining an age range for YOH was challenging as there are no specific guidelines for defining the term “young adult”. While the definition of 18–39 years is contextually relevant to young Malaysian adults, further examination and standardization of the definition of young adults in the global context is recommended, for example, those between the ages of 18 and 25 years.

### Summary

#### What is known about this topic

- Prospective epidemiological studies have shown that BP has a continuous, graded adverse effect on the risk of developing future cardiovascular events. In short, the earlier the onset of hypertension, the longer its duration, and the greater the risk for future cardiovascular events.
- Recognizing the importance of the health of young adults in relation to hypertension, some communities and countries have already begun examining YOH to highlight this issue in their local setting. In the USA alone, it has been elucidated that nearly one in five young adults have hypertension and measures are already underway to expound and address this.

#### What this study adds

- Utilising data from the NHMSs over 10 years from 2006 to 2015, this study reveals the pervasiveness of YOH in Malaysia. Data on trends of YOH are important to monitor the burden of cardiovascular outcomes, and assess the effectiveness of available public health interventions over time.
- Subgroup analysis reveals important target groups and focused areas on the management and prevention of hypertension among young adults.
